# Reframing cancer: challenging the discourse on cancer and cancer drugs—a Norwegian perspective: Reframing Cancer

**DOI:** 10.1186/s12910-021-00693-5

**Published:** 2021-09-21

**Authors:** Mille Sofie Stenmarck, Caroline Engen, Roger Strand

**Affiliations:** Centre for Cancer Biomarkers, Centre for the Study of the Sciences and the Humanities, University of Bergen, Bergen, Norway

**Keywords:** Cancer, Cancer treatment, Cancer drugs, Priority setting

## Abstract

**Background:**

As the range of therapeutic options in the field of oncology increases, so too does the strain on health care budgets. The imbalance between what is medically possible and financially feasible is frequently rendered as an issue of tragic choices, giving rise to public controversies around health care rationing.

**Main body:**

We analyse the Norwegian media discourse on expensive cancer drugs and identify four underlying premises: (1) Cancer drugs are de facto expensive, and one does not and should not question why. (2) Cancer drugs have an indubitable efficacy. (3) Any lifetime gained for a cancer patient is an absolute good, and (4) cancer patients and doctors own the truth about cancer. Applying a principle-based approach, we argue that these premises should be challenged on moral grounds. Within the Norwegian public discourse, however, the premises largely remain unchallenged due to what we find to be unjustified claims of moral superiority. We therefore explore alternative framings of the issue of expensive cancer drugs and discuss their potential to escape the predicament of tragic choices.

**Conclusions:**

In a media discourse that has seemingly stagnated, awareness of the framings within it is necessary in order to challenge the current tragic choices predicament the discourse finds itself in. In order to allow for a discourse not solely concerned with the issue of tragic choices, the premises that underlie it must be subjected to critical examination. As the field of oncology advances rapidly, we depend on a discussion of its opportunities and challenges that is meaningful, and that soberly addresses the future of cancer care—both its potential and its limits.

## Background

The field of oncology is characterised by rapid expansion of therapeutic options [1]. Novel treatment strategies are, however, costly and cancer drugs claim increasingly large proportions of national health care budgets. This has led to a situation characterised by an imbalance between medical opportunities and economic realities: The price of cancer treatment challenges healthcare systems’ capacity to provide ‘affordable, population-wide access to cancer medicines’ [2]. Within the frames of finite healthcare budgets, the principle of opportunity cost implies that prioritisation is necessary [3]. In line with priority-setting principles, accessibility of cancer drugs may consequently be denied when their costs outweigh provided benefit. Thus, the issue of health-rationing in relation to expensive cancer drugs has become a highly relevant and much-debated issue, both in public discourse as well as among health care providers and cancer researchers.

In the public as well as political and medical discourse, one of the dominant frames of the issue of expensive cancer drugs is that of tragic choices: suffering and death by cancer is an intolerable evil for the individual patients, while the drug prices are intolerable to society [4, p. 129]. This conflict of interests, and the polarising way in which the issue is presented, gives rise to controversies from which no sustainable solutions appear to emerge. The study presented in this paper was part of a larger research programme on the ethical and social aspects of cancer research and cancer care within the Norwegian centre of excellence *Centre for Cancer Biomarkers*, CCBIO. CCBIO was established in 2013 and throughout its existence it has observed and lived within a Norwegian context of never-ending public controversies around expensive cancer drugs. A recurring narrative, presented in hundreds of Norwegian newspaper articles, is that of cancer patients and their representatives experience of the government killing them by denying them access to novel therapeutic agents, over concerns for the health care budget. Such polarised—and arguably polarising—narratives, have become so typical of the debate that it has stagnated, with a continuous reproduction of virtually the same positions.

To us, this suggested there was a need for research on public media framings on the issue of priority-setting in relation to expensive cancer drugs, in order to understand the structure of the debate. This paper is composed of three parts. First, we briefly present the results of an empirical study of the Norwegian media debate. We outline the relationship between various identified frames and show how there is a striking lack of polyphony in the debate. We identify four assumptions, characterising the various frames, which remain unquestioned throughout the material we have analysed. Next, we systematically explore whether these assumptions are unquestionable from the perspective of medical ethics as well as social and political critique. Finally, in the third part we subject the same four assumptions to critical analysis. We further indicate how the results may contribute in moving thinking beyond that of tragic choices, pointing towards a reframing of cancer.

We wish to note that this study has focused on the discourse in Norway, and thus, on how the issue of cancer and cancer drugs presents itself there. As a contrast, the USA has the highest healthcare expenditure worldwide, estimated at over 18% of its GDP [5], whilst Norway, although another top spender, has a considerably lower health care expenditure with an estimated 10.2% of its GDP [6]. Further, the US has a healthcare system modelled on an entirely different design, and does not have universal healthcare coverage, as Norway does. It is perhaps best described as a hybrid system between federal, state and local governments, part national health services, part single-payer national health insurance, part multi-payer universal health insurance—to name some of the central components. This means that some of the issues central to the Norwegian debate on cancer drugs will not be issues in the US. The frameworks are different, because the models, and thus, the unique issues inherent to those models, differ. Similarly, many of the difficulties presented in the Norwegian discourse would appear entirely foreign to, for example, Somalia, which has one of the world’s lowest GDPs and faces a different set of healthcare issues altogether. For this reason, some of the premises uncovered in our study may be particular to Norway and the Norwegian discourse.

## Main text

### Setting the scene: public media framings in Norway

#### Material

The empirical material for this study was retrieved from the Norwegian newspaper database *Atekst.* This database comprises several thousand articles on the topic of cancer drugs and priority-setting issues. We selected the seven largest national newspapers (Aftenposten, Bergens Tidende, Dag og Tid, Dagbladet, Klassekampen, Morgenbladet and Verdens Gang) as a basis for our material, as they represent central actors in the debate. In addition, we included Norway’s largest and most influential newspaper for the health services (Dagens Medisin). We studied articles published from 2013 to 2017, as the debate became particularly relevant in 2013 following the admittance of ipilimumab for use in Norwegian public hospitals in the treatment of melanoma. Through exploratory research on the national database of all published newspaper articles, a set of keywords were selected which aimed to include all relevant articles to the debate and exclude those considered irrelevant. The keywords were as follows: “((kreft AND medisin*) OR kreftb* OR kreftm*) AND (priorit* OR nekt* OR Beslutningsforum*)”. We also specified the search to exclude the following words, as they led to an inclusion of a large number of articles not relevant to this particular debate: “respirator* OR xiaobo OR spinraza”. This search produced a total of 439 newspaper articles.

#### Framing theory

In this study, framing theory was applied to the debate on expensive cancer drugs and priority-setting dilemmas by considering how the various aspects of the issue had been contextualised by the media. The methodological approach we used is based on the work of Erving Goffman, who argued that there is no way of interpreting what we see, hear or read without subconsciously framing it in our minds, and thus giving it meaning. As such, frame analysis is a study of the cognitive organisation of experiences [7, p. 13]. Accordingly, we considered not only *what* is said in the media, but also *how* it is said, identifying the most common frames within the debate, and how these might affect the readers’ understanding of the issue. Each newspaper article and each identified frame was subsequently structurally examined in light of four major considerations: (1) what is the problem presented in the frame, (2) who are the actors, (3) who has the power, and (4) who are the sources.

#### Results and analysis

Applying framing theory and subjecting the material of 439 newspaper articles to close reading, we identified nine major frames [8]. They are summarised in Table 1.

**Table 1:** *Frame condensate*: The nine major frames of articles on cancer drugs and priority-setting issues in 8 largest Norwegian newspapers, 2013–2017

No.	Frame condensate
1	Suffering and desperate patients denied life-saving medicines
2	The unjust health system that sees patients as numbers
3	The slow and inefficient priority-setting system takes the lives of patients as they wait for expensive drugs to be approved
4	The priority-setting system polarises Norwegian patients into rich vs. poor
5	The marginalisation of the elderly as the priority-setting system disfavours them
6	Irresponsible politicians damage the credibility of our priority-setting system
7	The inefficient priority-setting system which delays approval of new drugs
8	The birth of the polarised health system
9	The priority-setting system gives power to individuals not suited to wield it

Though these various frames represent the breadth and variation of the debate, what was pervasive was their commonalities. In almost every frame the constellation of actors was the same; patient/doctor/patient organisation versus the priority-setting system/health authorities/politicians. These actors and the deliberate dichotomy between them were represented in a similar way in terms of distribution of power, with the former group presented as virtually powerless, and the latter as the powerful. Similarly, the sources were decidedly unvaried, and consisted mostly of patients, doctors and the Norwegian Cancer Society. Most importantly, the problem was invariably unvaried: a suffering cancer patient is denied a cancer drug, and this is inherently unjust. Thus, a pattern emerged of a conspicuous lack of variation across these frames. Following this observation, and as a consequence of a closer study of the frames, the analysis identified four major assumptions:

1. Cancer drugs are de facto expensive, and one does not and should not question why.
2. These cancer drugs work, and there is no need to question the validity of this claim.
3. Prolongment of life for a cancer patient is an absolute and unproblematic good, and any gained time—whatever shape or form that time has—is a blessing.
4. Patients, and their doctors, own the truth about cancer and cancer drugs, and “outsider” views on these are irrelevant, or unwelcome.

These four assumptions were so prevalent in the material, and the questioning of them so distinctly absent, that they arguably represent the premises which underlie the basis of the discourse on cancer and expensive cancer drugs in Norway in recent years. Further, this study considered articles produced over a period of four years—yet it was evident that, throughout these years, there was no sign of any meaningful progression. The same problems were presented time and again, with no real development emerging. To us, this demonstrated that the discourse is stagnant, and without consciously challenging the premises that underlie it, there is little evidence to suggest that this will change—and the issue of cancer and cancer drugs will thus remain one of tragic choices.

### Questioning the premises—is this ethically defensible?

Our study suggests that these four premises, ubiquitously present in the Norwegian debate on expensive cancer drugs and priority-setting, in effect monopolised the public discourse to a point where no other understandings of the debate could exist. In the following section we will argue, however, that the perceived tragic character of the issue of cancer and expensive cancer drugs to a large extent results from the adherence to these underlying premises, and the apparent unwillingness to challenge them.

Because challenging these premises is at the very best viewed as tactless, and at the worst considered genuinely unethical, we wish not only to discuss their substance but also briefly consider whether questioning them is unethical from the perspective of established norms within medical ethics. This is perhaps a slightly unusual exercise. From a philosophical perspective, attempts at rational argument are rarely (if ever) seen as something in need of ethical justification. However, in our material—as well as in our own experience with public debates in Norway on these issues—the four premises are often stated with a claim of moral superiority in the sense that it is implied that anyone who disagrees, is unethical and cold-hearted. They have become so well-established that arguing against them may put the questioner in such an unfavourable light that in this particular context it is a task no one seems willing to take upon them. We shall return to this later in the commentary.

Accordingly, we have chosen to spend some attention on the unusual exercise of reflecting on the moral status of the critique itself by discussing it against a well-known framework within the field of medical ethics, namely that presented by Beauchamp and Childress. In ‘Principles of Biomedical Ethics’ [9], the authors present four principles as a standard framework within the field, which are as follows: (1) *autonomy*, concerning the individual’s right to make decisions about their life and health, and the right to self-determination; (2) *beneficence*, one must act in the best interest of the other, in medical settings in terms of the patient; (3) *non-maleficence*, which is in accordance with the *primum non nocere*—“above all, do no harm”—principle of the Hippocratic Oath; and (4) *justice*, there must be equality among the individuals concerned, and the distribution of medical goods and services must be fair.

We will in the ensuing section critically examine the validity of each premise within the discourse, before applying the approach of the four principles to the questioning of said premises.

#### Premise 1: Cancer drugs are de facto expensive

The necessity of high pricing of novel cancer drugs was virtually unchallenged in our material. The market for medical technologies advances at a much higher growth rate than most GDPs. Though the high, and at times exorbitant, prices are problematised in the Norwegian discourse, it is the fact that they are so costly—not *why* they are so—which is considered problematic. Given the high prices of drugs and how this forces the health sector to make tough priority-setting decisions, it is interesting that it is still the unfairness of priority-setting itself rather than the costs that necessitate it, that remains the focus. One may argue that if one cannot afford the price of a drug, one should question not only why it is unaffordable—but also why the drug is sold at such a high price and why the pharmaceutical sector is regulated in such a manner. While it remains outside the scope of this commentary to review the many features of the market structure and business models of the pharmaceutical industry, there are several features that strengthen the pricing power of the producers. This includes monopoly or oligopoly for certain drugs; the fact that the end-users (doctors and patients) are normally not the paying customers; and the acceptance of practices in which pricing is not only unique to each customer, but also kept secret from other customers [10]. In sum, it is known that market prices are decoupled from R&D and production costs [1], and the pharmaceutical sector enjoys high profit margins—which can be linked to the loss of life through health care rationing within limited health care budgets. It surprised us that this point is not highlighted or problematised more in our material.

Having questioned the validity of the premise, let us briefly consider whether doing so is ethically defensible in line with the principles of *autonomy*, *beneficence*, *non-maleficence*, and *justice*. We believe so. Questioning whether cancer drugs must be expensive by critically exploring the mechanisms regulating the development, regulation and pricing of cancer drugs does not compromise patients’ *autonomy* nor does it infringe upon the principles of *beneficence* or *non-maleficence*. Indeed, a lively debate about the market structure and business models of the pharmaceutical industry might provide ideas for alternative and more sustainable and equitable models of drug development, regulation and pricing that also respect innovators’ and producers’ legitimate right to profit. Our argument here is simple and may sound trivial, and yet we were surprised to see an absence of such a debate.

#### Premise 2: New and expensive cancer drugs work

A recurring theme within the public discourse is that novel cancer drugs represent advancements towards the cure of cancer. Our findings suggest that novel cancer drugs are interpreted as a sign of medical progress. As such the efficacy of novel cancer agents remains largely unchallenged. These agents are often presented as a “last chance” therapy to those suffering from cancer [5, p. 130]. However, from an empirical perspective, the actual health gain resulting from such agents is known to be modest. Davies and colleagues performed a systematic evaluation of cancer drugs approved by the European Medicines Agency during the period 2009–2013. While some patients treated with these agents undoubtedly experienced durable and clinically meaningful responses, the results of this study suggested that evidence of clinical benefit was frequently lacking at time of approval and that on average the quantifiable benefits were marginal. The estimated median life expansion provided by these agents was modestly 2.7 months (Range:1–5.8 months) [11]. Lack of critical appraisal with regards to the efficacy and clinical utility of novel cancer drugs is a characteristic feature of the scientific discourse. Lancet oncology recently published a randomised clinical trial comparing the drug quizartinib (a tyrosine kinase inhibitor) and salvage chemotherapy, for the treatment of refractory, relapsed and unfit acute myeloid leukaemia. The trial demonstrated that patients treated with quizartinib gained an average of 1.5 months of life as compared to those on salvage chemotherapy. However, not only were these patients exposed to more treatment-related adverse events, such as septic shock, pneumonia and febrile neutropenia, but 33% of quizartinib patients were subject to treatment-emergent death, compared to 17 % of those offered traditional lines of therapy. Yet the conclusion of the study was that ‘treatment with quizartinib had a survival benefit versus salvage chemotherapy and had a manageable safety profile in patients with rapidly proliferative disease and very poor prognosis. Quizartinib could be considered a new standard of care’ [12, p. 986]. If these results represent the standard one holds new cancer drugs to, and the efficacy one expects of these new drugs, there is indeed very good reason to question the path we are on, and the future of cancer treatment. And this is, unfortunately, not a one-off case; in a recent, comprehensive study of misreporting of medical trials in major medical journals, the authors found ‘extensive evidence of misunderstanding about correct outcome reporting’ [13, p. 15]—which leads to misleading information concerning the advancements new cancer drugs represent, both to patients, clinicians and society.

By exploring the empirical foundation of novel cancer drugs it is clear that most novel cancer drugs represent only modest advancements at best and that many compounds, despite their high prices, provide no benefit at all with regards to improving or prolonging the lives of cancer patients.

What is the moral status of critically exploring this premise? We argue that if one uncritically accepts the efficacy of these drugs patients’ *autonomy* may be directly compromised. Decisions with regards to various treatments and care become ill-informed, and thus, informed consent loses its value. A similar argument can be made with regards to *beneficence, non-maleficence* and *justice*. Still, we are aware of the counter-argument, namely that criticism may be misunderstood in the public sphere and undermine hope and trust in medical science and a decrease in  public support to health care and the research that helps develop it. Perhaps more so than with most public issues, criticism of the efficacy of medicines should be delivered with nuance and attention to detail.

#### Premise 3: Any prolongment of life for a cancer patient is an absolute good

The third premise follows a similar logic to the second, with the assumption that any increase in the lifespan of a cancer patient is a victory, and indeed also the most important one. The public discourse does not seem to explore what a meaningful health gain comprises or whether there are limits to its value. Further, it does not explore or narrate alternative kinds of health gains—is the only measurable and valuable health gain prolongment of life, or could pain relief, time at home rather than at the hospital, and time with loved ones be more valuable? It is also neglectful of what this health gain *is*—what shape does the time these drugs offer patients, have, and what health states do novel cancer drugs provide? The material suggests that there is a prevailing conviction that the quantity of healthcare received—and the amount of cancer drug provided to each patient—is representative of the quality delivered. It seems that, within the healthcare system generally and with cancer specifically, the focus is on ‘how to make people live longer…but not on how to make lives mean more’ [14, p. 112]. The presentation of cancer management—both what this signifies for patients and families, and what it should include of health services—is grossly simplified in the discourse. In reality, cancer management concerns far more than eradicating illness; it is also, and at times more importantly, about ‘providing care, enabling the restoration of functions, self-worth, confidence, and motivation’ [15, p. 118]. Cancer is a multifaceted and complex illness—and so is the reality of suffering from it. We argue that by challenging the current—and century old—cultural perception of cancer as “the emperor of all maladies”, a more honest portrayal of the illness and its management might emerge. In order to meaningfully discuss the future of cancer drugs, we need a more nuanced debate not only in terms of how we handle dilemmas of priority-setting in relation to these drugs and their prices, but also in its approach to what cancer treatment itself is and should be.

We argue that questioning whether prolongation of life is an absolute good is not only ethical: it is essential. If patients are to maintain their *autonomy* throughout their illness, they should be well-informed about what their treatment options are, of what treatment entails, and of what that treatment could cost them. A public discourse which reflects the reality and scope of cancer treatment could further strengthen patient *autonomy*. The principles of *beneficence* and *non-maleficence* requires us not only to not cause needless pain, but to actively prevent it. If one is to maintain the principle of *primum non nocere*, one has an ethical obligation to consider whether these treatments are likely to provide health gains greater than the harm they cause [16, p. 55]. As Beauchamp and Childress argue, one cannot make judgements concerning medical benefit without considering the impact on quality of life [16, p. 150]. Finally, the principle of *justice* is central here for two reasons. First, it is not just to offer patients hope of prolonged life by providing treatment which could potentially make the precious time they have left stripped bare of any real quality. This may enhance suffering. Secondly, justice is served when the distribution of healthcare is fair; over-treatment, and treatment with drugs that provide small effect sizes at great costs, are unlikely to be considered fair, or reasonable, in any cost-benefit analysis. Often, these cancer drugs are the best alternative, both personally for the patient and financially for society, and can provide prolonged life with enhanced quality—but sometimes it is not, and we owe it to patients, and society, to not be afraid to ask the question: should we treat?

#### Premise 4: Patients and doctors own the truth about cancer and cancer drugs

Premise four, patients and doctors own the truth about cancer and cancer drugs, asserts that “outsider” views on these issues are irrelevant and unwelcome. We argue that the views of those suffering from cancer and of those who treat it are highly relevant to the debate and are thus of great importance. They do, however, also represent a  narrow set of viewpoints on the issue. Within a public media debate, listening to and quoting such a limited set of voices and opinions may restrict an open debate and hinder the exploration of the many issues within it, and as such impede progression and maturation. Further, if such a debate is to represent the concerns of the general public, it must include its respective viewpoints. Cancer is a disease so common that in some way or other—as patients, next of kin, healthcare personnel etc.—we all have experiences with it. As such we all have a stake in the debate and in the future of cancer care and treatment. One might also look beyond the issue of cancer and cancer treatment, and consider what the opportunity cost is in relation to other diseases and their treatments. When resources are directed towards cancer patients, they are denied somewhere—and to someone—else. This issue is complicated by the size and influence of the Norwegian Cancer Association, far stronger than representatives of many other patient groups. When this representative is given such a loud and powerful voice, we run the risk of allowing it to hush other representatives and other patient groups. We thus argue that the right to an opinion on the matter does not and should not be a prerogative reserved for cancer patients, their patient organisation, or oncologists.

Patients’ *autonomy* is not weakened by a public debate on cancer and cancer treatment generally. In some ways a broader representation of views could rather benefit them as their ability to make informed decisions with regards to treatment choices may improve by moving thinking beyond current conceptions of what cancer treatment is and could be. Further, if we agree that cancer is an issue that concerns us all it is both in our best interest (*beneficence*) and protects us from harm (*non-maleficence*) by allowing us to partake in discussions on the future of the illness, of treatment, and ultimately of our approach to this malady. Finally, there are arguably few ways to better protect the principle of *justice* than by allowing all to voice their opinions and partake in the discourse on an issue that so definitively concerns us all.

#### “Power of Goodness” and Moral Superiority

Above, we have argued that the underlying premises of the public discourse on cancer and cancer drugs—as exemplified by a Norwegian newspaper material—ought to be questioned and challenged.

Why, then, have these premises remained unchallenged? In order to pursue this question, we will take a sociological perspective and draw on the Norwegian sociologist Jill Loga’s analysis of the interface between morality and politics in the Norwegian public sphere [17]. In this study, Loga proposed the concept of *godhetsmakt* (in Norwegian; we have translated the term into “power of goodness”). She examines the effect professing goodness has on a discourse. She contends that ‘administering goodness can give individuals a privileged position to speak and act from’ [17, p. 321]. She argues that when an argument within a discourse openly claims to represent goodness, and fairness, that position is granted a superior stance against its opponent. Further, she argues that:

[…] open goodness has the ability to define its opponent. This creates a great discursive power. It becomes impossible to oppose against the open goodness, because one would appear as evil, cynical or egotistical. *If *one can administer and preach from the position of goodness, one becomes unimpeachable and immunized against criticism. Goodness needs only be expressed in order to become a conclusion [17, p. 323].

We argue that this is a central underlying mechanism in the public discourse on cancer and cancer drugs. Each premise, in its own way, claims a form of power of goodness in that they all supposedly speak to the cause of aiding suffering cancer patients, and thus, claim moral superiority. We can apply this to each premise: (1) cancer drugs are expensive, but that is not the point because they are worth it. Cancer patients must be saved. (2) Cancer drugs work, and there is no reason to question this because there is a need to believe that cancer patients can be saved. Questioning whether cancer drugs work indirectly questions whether cancer patients can be saved, which is too painful a question. (3) Any prolongment of life for a cancer patient is a good because it provides the illusion they are being saved. Drugs and prolonged survival is the only measurable benefit. And (4) patients and doctors own the truth about cancer, and this should not be questioned. That would be disloyal to them and would both undermine and enhance the suffering they are subjected to. Thus, it becomes good to uphold the premises, because they represent *goodness*.

Through claiming the power of goodness, certain stances gain discursive control. The position is powerful in that any opposition appears ruthless and cold. The claim of goodness gains moral incontestability, and a goodness discourse is therefore characterised by lack of polarity [17, p. 324]. Loga goes on to state that the lack of an opposing stance creates a self-reinforcing mechanism within the goodness discourse, and ‘discursive power forces one down a goodness spiral’ [17, p. 324]. That being said, not every position would make a successful claim of goodness. It must be seen as acceptable and reasonable within a larger social and cultural context and in alignment with core beliefs, norms and values; a prevailing discourse, as it were. Indeed, at the time of writing, in the midst of the COVID-19 pandemic, we could observe how the public discourse on public health changed from day to day, and with it, the acceptability of opinions and claims with regard to the proper measures to manage the pandemic. However, the material of our study covered a four-year period of public discourse on cancer drugs. Throughout this period—and arguably to this day—the premises remain unchallenged and their position uncontested. The goodness power they claim has stifled the discourse, with no solutions emerging, and with the issue persistently remaining one of tragic choices. These four premises have, through their goodness power, become four truths, and the public has accepted them as foregone conclusions. This demonstrates the incontestability the claim of goodness creates, and thus the ‘power of definition in the incontestable’ [17, p. 323].

In order to reframe the discourse on cancer and cancer drugs, allowing for a wider perspective and more truthful representation of these issues, it seems that one would need to challenge the set of deeper beliefs just presented by erecting alternative imaginations of the future. A full exploration is beyond the scope of this paper, but we will provide indications in the following part.

### Alternative imaginaries

Having presented arguments suggesting that the premises should be critically examined, we considered in part why the public discourse on cancer and cancer drugs is based on these premises, and why they have remained unchallenged—through the notion of “power of goodness”. The next step is to consider what the public discourse on the issue would resemble if we do indeed problematise the premises, and if this could alter the current perception of the issue as one exclusively of tragic choices.

As explained above, we shall do so by exploring alternative future imaginaries for the issue of expensive cancer drugs. The “deeper” beliefs presented in the preceding section provides us with the guiding principles for that search.

In terms of considering alternative imaginaries to the current discourse, and the predicament our healthcare system finds itself in, we will suggest three separate axes. We will not delve deeply into these, as such an endeavor requires a separate and comprehensive study, but we wish to suggest them as imaginaries that could alter the way we currently perceive the issue of cancer and cancer drugs, and the challenges this current perception has generated.

#### Axis I: Institutional change

We noted above how the claims to goodness rest on a certain blindness towards the institutional level, or possibly a lack of belief in the possibility of institutional change. This axis therefore ties into the first premise, namely that cancer drugs are de facto expensive and one need not—or should not—question why. History, however, demonstrates institutional fluidity. Healthcare delivery systems, whether based on universal healthcare or entirely dominated by private actors, are profoundly interrelated to both regulatory as well as policy dimensions. When drugs have become so expensive, this is a result of particular societal choices on how the health care sector, the industry and the pharmaceutical market have been organized and regulated. Globally, this sows inequality, and for affluent countries, the increasing prices increase the “health gap” between desirable and affordable health care. If this trend continues, one could end up with a system that is ‘managed not for the benefit of the people but rather for the corporations and the small elite who lead them’ [18, p. 236]. Further, all levels of income, health and illness ‘follow a social gradient: the lower the socioeconomic position, the worse the health’ [19, p. 1661]. As cancer drug prices soar, the population continues to age and medical opportunities increase, we are at a crossroads where the current healthcare system, and our understanding of sickness and health, has left cancer as an issue of tragic choices only. Institutional reconstruction is one way to force change on the tragic choices predicament of cancer. One could argue that the only way to make a genuine difference to public health today is to tackle *the causes of the causes*, with ‘major changes in social policies, economic arrangements, and political actions’ [19, p. 1668].

#### Axis II: Changing the public perception of disease and health

The claims to goodness seem to rest on a notion of health essentially linked to the prospects of cures for disease. Affixed to this notion is the second and third premise; that these cancer drugs have a definitive efficacy, and that any prolongment of life for a cancer patient is an absolute good. In modern society, as medical opportunities become ever greater, the decline of bodily functions, and the ultimate inevitability of death, seems to be presented as almost unnatural. This is mirrored in common cultural perceptions of what health is, where ‘health and disease are perceived as anchoring opposite ends of a continuum’ [20, p. 295]. Disease is all that health is not, and so it follows that any deviation from perfect health must be disease. One refuses to accept disease, because one expects to be saved from that state by the magic of modern medicine. Indeed, ‘our technological culture has lulled us into the false sense that health is a natural state and that medical intervention can repair any malfunction’ [20, p. 302]. Cancer, in particular, seems difficult to accept, because the image of cancer is one of pain and suffering. This view of health and disease is perhaps the result of modern life and the enormous technological leaps medicine has taken in the last century, and last decades. But no matter the miracles modern medicine seems capable of, we remain definitively mortal, and death is a certainty we cannot negotiate. One may argue that unwillingness to accept both disease and death may ultimately enhance suffering, in that death is inevitable. Thus, an obvious way of challenging cancer as an issue of tragic choices is to alter the view of it. Alternative imaginaries may emerge by envisioning changes in how individuals and societies relate to disease and health generally. Indeed, by broadening the view of health itself, one could potentially provide a legitimate place for cancer, and for disease generally, which is not dominated solely by suffering. As one cancer patient, stated, ‘I know I am ill… but I feel healthy’ [20, p. 301].

No society can provide every citizen with everything they want—yet it seems, from the public discourse and the premises that underpin it, that this is what is expected when discussing disease, specifically and especially in relation to cancer. The four premises, and the debate they have given birth to, arguably maintain unrealistic and unachievable expectations of what illness and health are and should be. The material suggests that the discourse is concerned solely with eradicating disease, and that the ultimate goal for every patient, every clinician and every society is to prolong life as much as medically and biologically possible. The question is whether this really is what cancer patients and society wants? Brekke and Sirnes’ [21] “hypersomatic individual” illustrates this development. With both increased information regarding disease as well as a violent fear of cancer in general, this individual’s desperation pushes the limits and promises of science, and pursues a continued alienation of the prospect of suffering and death. As Brekke and Sirnes write, ‘the ambitions and fantasies of biomedical science…are not only taken seriously by this individual, but are also transformed into a combination of an action program and a list of quasi-juridical demands for both science and politics’ [21, p. 358]. Arthur Frank’s theory of how the “scarcity loop” dominates modern healthcare describes a similar problem. He points to ‘the endemic contradiction in health care between the hopes, desires and expectations that capitalist techno-science thrives on generating, and the realities of what can be delivered and who can afford what’ [22, p. 24].

#### Axis III: Thanks, but no thanks

A final axis, and alternative imaginary, is rather alternative indeed—but nonetheless worth exploring. One can perhaps imagine a point along this path where patients, healthcare providers and society come to the conclusion that these treatments are so costly, their efficacy often so marginal and the suffering they expose patients to so great that it simply is not worth it anymore—the benefit is too little, and the opportunity cost too high. This axis corresponds with the fourth premise, that cancer patients and their doctors own the truth about cancer and cancer drugs. In this imaginary, both the narrator and the narrative on cancer is altered, and understandings of how to meet disease and treatment in the future differs drastically from the current climate—at least pre-COVID-19 and the potential effect the pandemic might have on public health policies and the economic system that ultimately funds the healthcare system.

What would that world look like? Perhaps one where cancer management, in the advanced stages, is more concerned with care and support of patients, rather than treatment of tumors; where knowledge production and the development of novel health care practices aim at enriching and empowering the remaining life of cancer patients rather than prolonging it at any cost. This may result in the emergence of novel, non-pharmacological tools that could provide relief of suffering for cancer patients and their loved ones. One needn’t remove the treatment options we already have, but one might choose not to spend public resources on drugs of whose potential little is known, but whose cost is all too familiar. More resources could alternatively be spent on managing symptoms and supporting and accommodating the psychological and existential needs of cancer patients and their loved ones. In such an imaginary, treatment outcomes may not become any worse; what is sacrificed, is the hope of technoscience and big pharma delivering the imminent cure. The result could very well be the emergence of a society that more profoundly acknowledges the boundary conditions of human life: death. It is not unimaginable that comfort and relief of suffering could be found by coming to terms with life’s finiteness. This imaginary is arguably far removed from the current climate on illness and disease, where healthism seems to play a central role, as originally analysed and critiqued by Robert Crawford [23] and Petr Skrabanek [24]. Good health is taken as an absolute good, incommensurable with other values, or rather infinitely higher than other values. This imaginary may therefore indeed be radical; if so, it is evidence of how deeply entrenched the idea of healthism has become, almost a full generation after Crawford and Skrabanek.

## Conclusions

If the issue of cancer and expensive cancer drugs is to develop into something more than an issue *solely* of tragic choices, we argue that each of the premises of the public discourse today as we have characterised it, should be subjected to critical examination, empirically and normatively. We argue that questioning the premises opens for a much-needed reframing of the debate. We considered alternative imaginaries as a way of demonstrating that the current framework can and should be challenged, and that doing so can stimulate a more open debate, and potentially liberate a stagnant discourse.

As stated earlier, the above-mentioned imaginaries are precisely that—imaginaries—and we do not claim to provide solutions to the dilemmas born from expensive cancer drugs. Indeed, this discussion is not about identifying the ideal future of cancer drugs and cancer treatment. We do, however, believe that awareness of issue framings could have a wider impact in terms of approaches to the healthcare system, to treatment options, to illness and ultimately to health. In order to tackle the challenges the future of cancer treatment presents, one needs a public discourse which includes a meaningful discussion on what illness and health ultimately is, which health states one should strive for and what one can reasonably expect from standard care. A promising way forward could be to ask what the core problem is: we struggle to accept that some patients are denied certain treatments, so is it the injustice of this we object to? Is it the fact that so many are dying from one disease, is it the disease itself, or is it the way people die from it? Once the frame of tragic choices is challenged, the public discourse becomes better positioned to explore and discuss these questions.

## Data Availability

The newspaper data analysed were retrieved from Atekst, which is a commercial service owned and operated by the Retriever Group (https://www.retrievergroup.com/ ). Accordingly, the data are not granted open access but subject to license.

## Abbreviations

CCBIO: Centre for Cancer Biomarkers, University of Bergen, Norway
COVID-19: Corona virus disease 2019
GDP: Gross domestic product
QALY: Quality-adjusted life year
R&D: Research and development
US/USA: United States of America
